# Linear Darier disease treated with ruxolitinib: Case report and review of the literature

**DOI:** 10.1016/j.jdcr.2026.03.054

**Published:** 2026-04-09

**Authors:** Jamie Warner Kahli, Patricia Martin, Tara Ezzell

**Affiliations:** a University of Florida College of Medicine, Gainesville, Florida; b Burrell College of Osteopathic Medicine, Melbourne, Florida; c Dermatology Associates, Gainesville, Florida

**Keywords:** ATP2A2-nEDD, case report, Darier disease, dermatopathology, genetics, genodermatosis, itch, JAK inhibitor, immunodermatology, keratosis follicularis, linear Darier disease, mosaicism, pruritus, ruxolitinib

## Introduction

Darier disease, also known as ATP2A2-nEDD (nonsyndromic epidermal differentiation disorder) or historically as keratosis follicularis, is a rare autosomal dominant genodermatosis caused by mutations in the ATP2A2 gene, encoding a calcium pump within the endoplasmic reticulum.^1^ Classic Darier disease is characterized by a diffuse eruption of keratotic papules and the development of longitudinal erythronychia.^2^ The papules are indurated and may be skin-colored, yellow-brown, or brown.^3^ Affected sites include seborrheic areas of the face, scalp, neck, trunk, elbows, and skinfolds; papules can also arise inside the mouth.^3^ Several papules can coalesce into plaques, which may develop an odor, especially if located in skinfolds.^3^ The lesions of Darier disease are often colonized with *Staphylococcus aureus*, *Corynebacterium*, *Propionibacterium*, and *Paracoccus* and can become infected, further contributing to malodor and discomfort.^4^ Other signs and symptoms include pruritus exacerbated by heat and friction, pitting of the palms and soles, and ecchymosis.^3^

The estimated prevalence of Darier disease is 1 per 100,000 people, and it presents predominantly in patients between 15 and 30 years old, with no gender or ethnicity-based predilection.^2^ A rare form of Darier disease known as linear form, or acantholytic dyskeratotic epidermal nevus, is characterized by keratotic papules that follow Blaschko’s lines, characterizing cutaneous mosaicism.^3^ Lesions in linear Darier disease are not as widespread as in classic Darier disease.^3^ Some individuals with the linear form of the disease also have the nail abnormalities (erythronychia) seen in those with the classic form, but only unilaterally.^2^ Histology of Darier disease shows focal acantholytic dyskeratosis with papillomatosis.^3^ Features include corps ronds, seen as dyskeratosis, apoptosis, and acantholysis.^3^ Linear Darier disease is histologically similar to classic Darier disease, differing only phenotypically by following its distinct linear dermatomal distribution pattern.

## Case report

**Fig 1:**
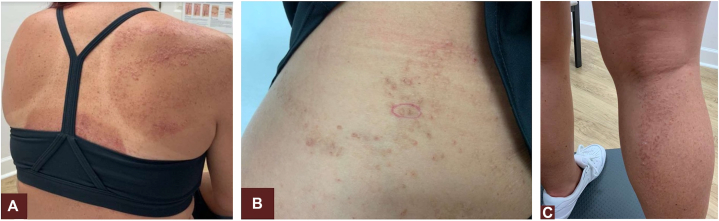
Pretreatment appearance of lesions. At presentation, the patient’s right shoulder **(A)**, lower portion of the right side of abdomen with the biopsy site marked **(B)**, and right leg **(C)** demonstrated skin-colored hyperkeratotic papules following the Blaschko lines.

A 31-year-old White woman with no significant medical or surgical history and taking no medications presented with a pruritic rash with hyperkeratotic skin-colored papules and plaques in a swirling pattern only on the right side of her body, which had waxed and waned since age 18 (Fig 1). She had no nail involvement. Her pruritus was exacerbated by heat and friction, and a gluten-free diet provided no improvement. The patient denies a family history of similar lesions, although no family members were present for us to look for subtle signs of prior lesions. Over several years, multiple topical treatments were trialed, including selenium sulfide shampoo, desonide 0.05% ointment, tacrolimus 0.01% ointment, and lactic acid 10% lotion with varying degrees of symptomatic relief but without significant clinical or cosmetic improvement. Given her longstanding history and multiple treatment trials, she consented to a shave biopsy of a 0.8 cm papule on the right side of her abdomen. The following empirical trial was followed while awaiting biopsy results: ruxolitinib cream 1.5% once daily to the right shoulder, ammonium lactate 12% lotion daily to the right side of the abdomen, and tazarotene 0.045% lotion daily to the right leg. Once-daily use of ruxolitinib cream 1.5% was selected because the patient was having to use samples; she could not afford the medication because insurance would not approve it until other treatments had failed.

**Fig 2:**
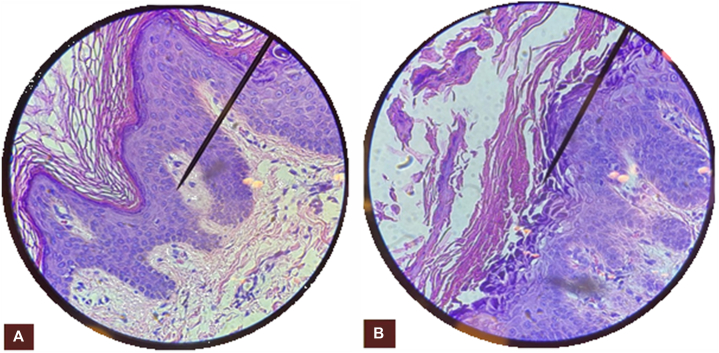
Hematoxylin and eosin–stained photos of pathology slides of a shave biopsy of the right side of the abdomen. Specimen slides showed papillomatosis **(A)** with acantholytic dyskeratosis **(B)** and corps ronds and grains, seen in Darier disease. (Original magnifications: **A,** ×400; **B,** ×400.)

Biopsy showed papillomatosis with invaginated areas demonstrating superficial acantholysis with corps ronds and grains (Fig 2). Histopathologic considerations included both Grover disease and Darier disease. Molecular genetic testing for ATP2A2 mutations was offered, but the patient declined for personal reasons. However, in view of the multiple papules and plaques in Blaschko’s lines and the characteristic histopathologic findings, clinical pathologic correlation led to the diagnosis of linear Darier disease.

**Fig 3:**
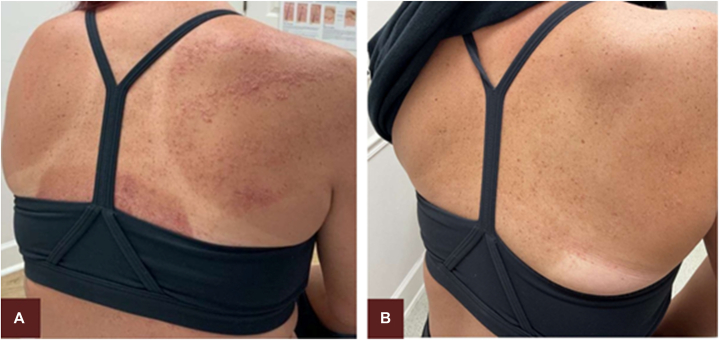
Patient’s back is shown prior to 6-week treatment with ruxolitinib cream 1.5% **(A)** with complete clearance of lesions, as well as pruritus, after 6 weeks of ruxolitinib on the right shoulder **(B)**.

**Fig 4:**
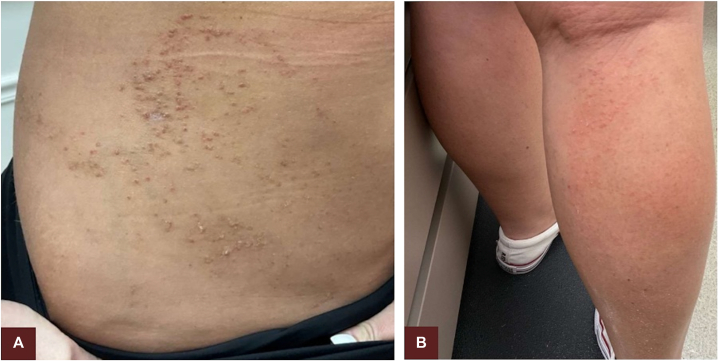
Incomplete clearance after 6 weeks of a keratolytic on the right side of the abdomen and a retinoid on the calf of her right leg. Ammonium lactate cream 12% on the right side of the abdomen yielded little improvement in the appearance of lesions and unchanged pruritus **(A)**. With tazarotene cream 0.045% on the right leg, the patient had a moderate decrease in the number of papules and the amount of scale on each papule but experienced xerosis and unchanged pruritus **(B)**.

After 6 weeks of use, the right shoulder treated with once-daily ruxolitinib cream 1.5% was completely cleared (Fig 3). The right side of the abdomen treated with ammonium lactate 12% had little improvement in the appearance of lesions and unchanged pruritus. The right leg treated with tazarotene 0.045% lotion moderately improved, with only scattered small scaly papules remaining on the calf, but with undesirable side effects of xerosis, pruritus, and erythema (Fig 4). Clearly due to superior disease control with ruxolitinib, the patient was instructed to discontinue all other treatments and apply only ruxolitinib to all affected areas, including the calf of her right leg and right side of the trunk. Over the phone, the patient reported that the lesions on the right side of her back and abdomen had resolved after 2 months of twice-daily ruxolitinib application and that the calf of her right leg had remained clear without any maintenance application of ruxolitinib. She noted no side effects from ruxolitinib other than postinflammatory hyperpigmentation, for which she was reassured that it would resolve with time. The patient was prescribed ruxolitinib cream 1.5% twice daily to affected areas as needed for flares, and she has remained completely clear for >12 months and counting.

## Discussion

Topical ruxolitinib cream 1.5% is a topical selective Janus kinase (JAK) inhibitor approved by the United States Food and Drug Administration for short-term treatment of atopic dermatitis in nonimmunocompromised patients aged 12 years and older whose disease is not controlled with topical therapies or when other topicals are not advisable.^5^ Ruxolitinib works by inhibiting JAK1 and JAK2, which are involved in several cytokine pathways, including the JAK-signal transducer and activator of transcription signaling pathway, which contributes to inflammation, pruritus, and skin barrier dysfunction.^5^ Reducing the activity of JAK1 and JAK2 has led to decreased severity of signs and symptoms of atopic dermatitis by reducing erythema and pruritus, with individuals reporting less itch and improvement in sleep quality.^5^

There is currently no United States Food and Drug Administration-approved therapy for Darier disease, and ruxolitinib, used in the treatment of Darier disease, has not been documented. JAK inhibitors have also been discussed as a promising tool for dermatologic conditions such as lichen planus and psoriasis.^5^ Systemic retinoids such as isotretinoin are first-line therapies for classic Darier disease, but given the localized distribution of linear Darier disease, topical treatment is generally advised.^6^ Topical tretinoin and keratolytics are used to reduce hyperkeratosis, as well as topical corticosteroids and immunosuppressants.^6^

Our patient had moderate improvement with topical retinoids, but she experienced erythema, xerosis, and pruritus, causing discomfort. Ruxolitinib was the only medication to show sustained clearance (>12 months and counting) without adverse effects. Darier disease is known to worsen in summer and improve in winter, as occurred with this patient, but the patient’s greatest improvement occurred between July and September, upon initiation of ruxolitinib. She remained clear at the most recent follow-up in the summer of the following year. To our knowledge, this is the first case of ruxolitinib being used in the treatment of Darier disease found in the literature at the time of publication.

Linear Darier disease is a rare genodermatosis that can present subtly and intermittently. Few effective treatments have been identified, and no United States Food and Drug Administration-approved medications exist for Darier disease. Topical ruxolitinib yielded promising results in this case report. Larger-scale trials are needed in order to determine reproducible results.

## Conflict of interest

None disclosed.
